# Efficacy of Mindfulness-Based Cognitive Training in Surgery: Additional Analysis of the Mindful Surgeon Pilot Randomized Clinical Trial

**DOI:** 10.1001/jamanetworkopen.2019.4108

**Published:** 2019-05-24

**Authors:** Carter C. Lebares, Ekaterina V. Guvva, Maria Olaru, Leo P. Sugrue, Adam M. Staffaroni, Kevin L. Delucchi, Joel H. Kramer, Nancy L. Ascher, Hobart W. Harris

**Affiliations:** 1 Department of Surgery, University of California, San Francisco; 2 Department of Neuroradiology, University of California, San Francisco; 3 Department of Neurology, University of California, San Francisco

## Abstract

**Importance:**

Mindfulness meditation training has been shown to be feasible in surgical trainees, but affective, cognitive, and performance benefits seen in other high-stress populations have yet to be evaluated.

**Objective:**

To explore potential benefits to stress, cognition, and performance in postgraduate year 1 (PGY-1) surgery residents receiving modified mindfulness-based stress reduction (modMBSR).

**Design, Setting, and Participants:**

This follow-up study is an analysis of the Mindful Surgeon pilot randomized clinical trial of modMBSR (n = 12) vs an active control (n = 9), evaluated at baseline (T1), postintervention (T2), and 1 year (T3), took place at an academic medical center residency training program among PGY-1 surgery residents. Data were collected between June 2016 and June 2017 and analyzed from June 2017 to December 2017.

**Interventions:**

Weekly 2-hour modMBSR classes and 20 minutes of daily home practice during an 8-week period vs an active control (different content, same structure).

**Main Outcomes and Measures:**

Preliminary evidence of efficacy was explored, primarily focusing on perceived stress and executive function and secondarily on burnout, depression, motor skill performance, and changes in blood oxygen level–dependent functional neuroimaging during an emotion regulation task. Group mean scores were calculated at T1, T2, and T3 and in linear mixed-effects multivariate analysis. Effect size for analysis of covariance is presented as partial η^2^ with the following cutoff points: small, less than 0.06; medium, 0.06 to 0.14; large, greater than 0.14.

**Results:**

Postgraduate year 1 surgery residents (N = 21; 8 [38%] women) were randomized to a modMBSR arm (n = 12) or an active control arm (n = 9). Linear mixed-effects modeling revealed differences at T2 and T3 in perceived stress (mean [SD] difference at T2: modMBSR, 1.42 [5.74]; control, 3.44 [6.71]; η^2^ = 0.07; mean [SD] difference at T3: modMBSR, 1.00 [4.18]; control, 1.33 [4.69]; η^2^ = 0.09) and in mindfulness (mean [SD] difference at T2: modMBSR, 3.08 [3.63]; control, 1.56 [4.28]; η^2^ = 0.13; mean [SD] difference at T3: modMBSR, 2.17 [3.66]; control, −0.11 [6.19]; η^2^ = 0.15). Burnout at T2 (mean [SD] difference: modMBSR, 4.50 [9.08]; control, 3.44 [6.71]; η^2^ = 0.01) and T3 (mean [SD] difference: modMBSR, 5.50 [9.96]; control, 5.56 [9.69]; η^2^ = 0.01) showed similar increase in both groups. Working memory increased more at T2 in the modMBSR arm (mean [SD] difference, 0.35 [0.60]) than in the control arm (mean [SD] difference, 0.21 [0.74]; η^2^ = 0.02) and at T3 (modMBSR, 0.68 [0.69]; control, 0.26 [0.58]; η^2^ = 0.20). Cognitive control decreased more in the control arm at T2 (mean [SD] difference at T2: modMBSR, 0.15 [0.40]; control, −0.07 [0.32]; η^2^ = 0.13) and at T3 (mean [SD] difference: modMBSR, 0.07 [0.59]; control, −0.26 [0.53]; η^2^ = 0.16). Mean (SD) circle-cutting time improved more at T2 in the modMBSR arm (−24.08 [63.00] seconds) than in the control arm (−4.22 [112.94] seconds; η^2^ = 0.23) and at T3 in the modMBSR arm (−4.83 [77.94] seconds) than in the control arm (11.67 [145.17] seconds; η^2 =^ 0.13). Blood oxygen level–dependent functional neuroimaging during an emotional regulation task showed unique postintervention activity in the modMBSR arm in areas associated with executive function control (dorsolateral prefrontal cortex) and self-awareness (precuneus).

**Conclusions and Relevance:**

In this pilot randomized clinical trial, modMBSR in PGY-1 surgery residents showed potential benefits to well-being and executive function, suggesting a powerful role for mindfulness-based cognitive training to support resident well-being and performance, as mandated by the Accreditation Council for Graduate Medical Education.

**Trial Registration:**

ClinicalTrials.gov identifier: NCT03141190

## Key Points

**Question:**

Is there preliminary evidence to support the effectiveness of mindfulness-based stress reduction for improving well-being and performance in postgraduate year 1 surgery residents?

**Findings:**

In this pilot randomized clinical trial of 21 first-year surgery residents, taking a modified mindfulness-based stress reduction class was associated with higher mindfulness, lower stress, better executive function scores, faster motor skills, and unique activation of neural substrates associated with executive control and self-awareness during an emotional regulation task compared with control participants.

**Meaning:**

Mindfulness-based stress reduction appeared to mitigate stress and enhance executive function in surgery residents, supporting the value of larger, more definitive trials of this promising intervention for surgeons.

## Teaser

This follow-up analysis of pilot randomized clinical trial data explores the potential benefits of mindfulness-based stress reduction for improving well-being among postgraduate year 1 surgery residents.

## Introduction

Among physicians, overwhelming stress has been linked to burnout, distress, depression, and suicidality^1^ and, in the absence of adequate coping skills, has been posited to promote performance deficits, from surgical errors to poor professionalism.^2,3,4,5^ While interventions to address work climate and work-life balance are growing,^6^ individual-based interventions that successfully mitigate the effects of stress remain scarce.^7,8^ This gap is particularly striking in light of a growing consensus that physician well-being is a cornerstone of sustainable health care that is hindered by overwhelming stress and equally dependent on institutional, systemic, and individual efforts.^9,10^

Resilience is a means of adaptive coping that changes perceived stress through the development of specific cognitive habits.^11,12,13^ Mindfulness-based interventions (MBIs) have been shown to enhance resilience^14^ and improve affect,^15,16^ executive function,^17,18,19,20^ and performance^21,22^ in other high-stress populations, such as Marines,^14,17^ police special forces,^23^ and elite athletes,^24,25^ suggesting the potential of MBIs to serve as stress resilience training for physicians. Mindfulness meditation training involves the cultivation of key cognitive skills, including the moment-to-moment awareness of thoughts, emotions, and sensations (ie, interoception),^26,27^ the development of nonreactivity in response to stimuli (ie, emotional regulation),^28^ and the conscious awareness of cognitive control processes (ie, metacognition).^20,29,30^

The most scientifically studied form of mindfulness training is the secular mindfulness-based stress reduction (MBSR),^29^ which is trained through an 8-week codified curriculum. Mindfulness-based stress reduction and other MBIs may work by strengthening an individual’s ability to tolerate uncomfortable experiences through training in nonreactivity to difficult thoughts, events, and emotions. Such nonreactivity may reduce the magnitude of perceived stress, which theory and empirical work suggest is stimulating to a point, after which impairment occurs.^31,32^ Working memory capacity (WMC), a primary domain of executive function and a key measure of cognitive load,^33^ is worsened by overwhelming stress.^17^ Thus, reducing perceived stress may reduce cognitive load, leading to improved WMC and executive function for complex tasks, such as clinical reasoning and surgical judgement—highly relevant skills for physicians and surgeons.

Despite evidence that MBIs enhance stress resilience, well-being, and performance in quantifiable ways, they have been little used in physicians. To address this gap, we first conducted a pilot randomized clinical trial of modified MBSR (modMBSR) in surgery interns at a US academic center and reported it to be feasible and acceptable.^34^ Here, we report on additional analysis of data from that trial, exploring preliminary evidence of MBSR efficacy regarding improved stress resilience, well-being, and performance.

## Methods

### Trial Overview

**Figure 1.**
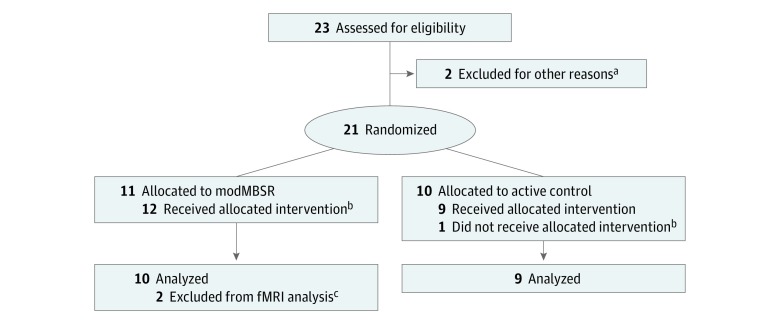
CONSORT Flow Diagram ^a^Two participants were enrolled but were withdrawn by their parent program before completing assessment battery or attending any study sessions owing to conflicts with specialty-specific didactic sessions and concern for compromised education. ^b^One participant was initially allocated to the active control but did not receive the intervention owing to inadvertently attending the modMBSR training class during week 1. She was therefore reassigned to the modMBSR intervention group. ^c^Two participants did not have functional magnetic resonance imaging (fMRI) scans analyzed. One was never scanned owing to implanted metal, and the other was scanned but data were incomplete (protocol glitch) and could not be analyzed.

The trial design, intervention, and feasibility findings have been described in detail elsewhere.^34^ Briefly, in 2016, we conducted a pilot parallel-group randomized clinical trial,^34^ Mindful Surgeon, with 1:1 allocation to modMBSR, consisting of 8 weekly, 2-hour classes,^34^ vs an active control (different content, same structure) in 21 first-year surgery residents (Figure 1). The study protocol and statistical analysis plan are available in Supplement 1. All aspects of the intervention and assessment were approved in full by the University of California, San Francisco (UCSF) institutional review board. Eligible participants were postgraduate year 1 (PGY-1) surgery residents at UCSF, without a current mindfulness meditation practice who provided written and oral informed consent and were blinded to assignment. They received no financial compensation. All data were collected at the UCSF Sandler Neurosciences Center, and deidentified data were analyzed by a biostatistician (K.L.D.). In line with the National Institutes of Health Stage Model for behavioral intervention development,^35^ trial goals were to show intervention feasibility and acceptability (reported elsewhere)^34^ and to generate preliminary evidence of MBI efficacy (ie, effect size) as well as feasibility of study methods in preparation for an adequately powered future trial. Thus, this study was not powered to detect statistically significant intergroup differences and comprises a sample of convenience. This study followed the Consolidated Standards of Reporting Trials (CONSORT) reporting guideline.

**Figure 2.**
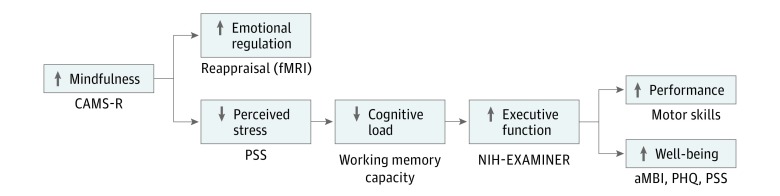
Working Conceptual Model and Associated Outcome Measures aMBI indicates abbreviated Maslach Burnout Inventory; CAMS-R, Cognitive Affective Mindfulness Scale–Revised; fMRI, functional magnetic resonance imaging; NIH-EXAMINER, National Institutes of Health Executive Abilities: Measures and Instruments for Neurobehavioral Evaluation and Research; PHQ, Patient Health Questionnaire; PSS, Perceived Stress Scale.

Our working conceptual model (Figure 2) is that MBI training enhances the development of cognitive habits that change how interns experience and respond to discomfort (ie, stress), thereby decreasing the magnitude of perceived stress and its contribution to cognitive load. Thus, available executive function resources (such as emotional regulation, WMC, and executive control) will increase and contribute to better mood, cognition, and performance.

### Measuring Efficacy

The primary efficacy outcome was change in perceived stress. The secondary outcome was change in executive function. Additional prespecified outcomes were burnout, depression, mindfulness, resilience, grit, motor skill performance, and changes in functional neuroimaging during an emotion regulation task. Outcomes were assessed at baseline (T1, before the start of intern year), postintervention (T2, 3.5 months after baseline), and at the end of the intern year (T3, 12 months after baseline).

#### Psychological Assessment

Participants completed an online psychologic survey consisting of reliable and published questionnaires shown to be sensitive in our prior work.^1^ The questionnaires included the Block Ego-Resilience scale,^36,37^ Cognitive and Affective Mindfulness Scale–Revised,^38^ Short Grit Scale,^39^ Perceived Stress Scale,^40^ abbreviated Maslach Burnout Inventory,^41^ and 9-item Patient Health Questionnaire (PHQ-9).^42^

#### Executive Function Testing

To evaluate the effects of modMBSR and medical training on executive function, we used the computer-based and paper-based National Institutes of Health Executive Abilities: Measures and Instruments for Neurobehavioral Evaluation and Research (NIH-EXAMINER),^43^ which comprises tasks targeting 6 cognitive domains (ie, working memory, inhibition, set shifting, fluency, insight, and planning) believed to subserve higher-order cognitive function, such as decision making and problem solving. The NIH-EXAMINER was developed to avoid ceiling effects, maintain validity within multiple demographic groups, and be suitable for repeated administration in clinical trials.

#### Motor Skills Testing

Motor skills were evaluated with 2 tasks taken from Fundamentals of Laparoscopic Surgery—peg transfer and circle cutting—scored for time and accuracy. The Fundamentals of Laparoscopic Surgery is a validated laparoscopic skills test developed by the Society for Advanced Gastrointestinal Endoscopic Surgery.^44^

#### Emotional Regulation Task

To explore changes in neural substrates associated with our intervention, we used a task of emotional regulation in the form of cognitive reappraisal^45^ (ie, the reinterpretation of affective stimuli to alter emotional impact). During a blood oxygen level–dependent functional magnetic resonance imaging (fMRI) scan, participants viewed aversive images selected from the International Affective Picture System.^46^ Images were displayed on a monitor in the scanner suite and viewed through a system of back-projecting mirrors mounted to the head coil unit. Participants viewed each image once (eAppendix 1 in Supplement 2).

**Figure 3.**
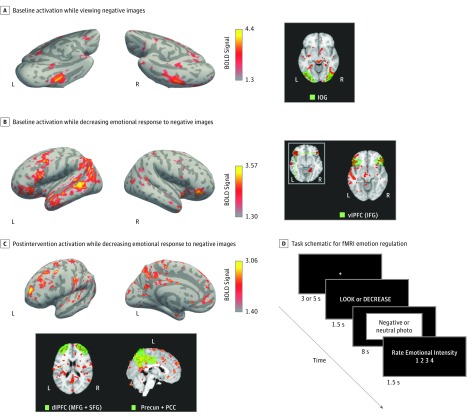
Functional Brain Scan Activation During Emotional Regulation Task A, Patterns of activation unique to viewing negative images in the intervention and control groups at baseline. Right panel shows activation of inferior occipital gyrus (IOG), which is associated with the processing of emotionally salient images. B, Patterns of activation unique to the action of decreasing emotional response to negative images (ie, reappraisal) in the intervention and control groups at baseline. Right panel shows activation of ventrolateral prefrontal cortex (vlPFC), which includes the inferior frontal gyrus (IFG) and is associated with the reinterpretation of affective stimuli to alter the emotional impact. C, Patterns of activation unique to the action of decreasing emotional response to negative images, only seen in the intervention group and only seen after the intervention. Lower panel shows activation of dorsolateral prefrontal cortex (dlPFC), which includes the middle frontal gyrus (MFG) and superior frontal gyrus (SFG) and is associated with the functioning of the executive control hub of higher-order cognition. The precuneus (precun), which is anatomically within the posterior cingulate cortex (PCC), is associated with mental imagery, visuospatial motor skills, and self-awareness. Both areas showed activation in the modified mindfulness-based stress reduction arm postintervention. D, Schematic diagram of the timing and steps involved in the presentation of each image for the emotional regulation task. BOLD indicates blood oxygen level–dependent; fMRI, functional magnetic resonance imaging; L, left; and R, right.

Images were grouped into 3 conditions. For the neutral and negative (ie, aversive) conditions, participants were instructed to view the image, understand it, and experience resultant feelings naturally. For the decrease negative condition, participants viewed negative images and were asked to decrease the intensity of their emotional response. All participants saw the same 60 images, in 3 sets of 20, delivered in the same fashion (Figure 3D).

### Statistical Analysis

For tests and surveys, group mean scores and SDs were calculated at 3 points and evaluated using *t* tests and Pearson χ^2^ tests. Consistent with recommendations for pilot trials,^47^ we did not focus on statistical power but did use linear mixed-effects modeling (analysis of covariance) for multivariate analysis, with baseline scores as a covariate, calculating effect size (partial η^2^) with 3 suggested cutoff points: small, less than 0.06; medium, 0.06 to 0.14; and large, greater than 0.14.^48,49^ Of relevance to future trials, power calculations suggest that a sample size of 40 participants in a 2-group comparison will have 80% power to detect an effect size expressed as partial η^2^ of 0.17. This is considered a large-sized effect and is approximately equivalent to a Cohen *d *of 0.91.

Imaging analysis was limited to 19 participants (10 from the modMBSR arm and 9 from the control arm). After standard preprocessing steps (eAppendix 2 in Supplement 2), fMRI blood oxygen level–dependent responses from the 8-second image-presentation period of each trial were analyzed using a general linear model with an event-related design fit to the 3 pseudorandomly occurring image types: neutral, negative, and decrease negative. Two comparisons, negative vs neutral and decrease negative vs negative, were used to test for activations related to the viewing of negative images and the process of decreasing negative emotions, respectively. We used a fixed-effects model to combine data across runs within a scanning session and mixed-effects models to perform higher-level within-group and between-group analyses, producing group-averaged whole-brain *z *statistical maps of contrasts in MNI 152 standard space (Montreal Neurological Institute).^50^ We used longitudinal analysis with a paired within-individual model to assess for training-induced changes in activation for each participant and groups between scans at T1 and T2.

We performed whole-brain and region-of-interest (ROI) analyses based on predefined networks of interest and networks showing unique activation in whole-brain analysis. Group results from whole-brain analysis are presented as raw *z *statistical maps to show that the patterns of task activation are robust and regionally specific (Figure 3A-C). Regions of interest were defined anatomically based on the Automated Anatomic Labeling atlas of the standard Montreal Neurological Institute brain template,^51^ with activations measured using the Featquery tool (FMRIB Software Library) and averaged across the 2 hemispheres. We used 1-sided 1-sample *t* tests to validate findings of activation during emotional reappraisal^52,53^ and 1-sided 2-sample *t* tests to assess the effect of modMBSR on reappraisal-related activation in the intervention and control groups, separately. Statistical significance was set at *P* < .05, and all statistical analyses were performed on R version 3.3.3 (The R Foundation).

To demonstrate task validity, we compared negative vs neutral image responses at T1 across all participants (Figure 3A), focusing on the inferior occipital gyrus, which is involved in the processing of emotionally salient images.^52,53^ To demonstrate task fidelity, we evaluated activation during the act of decreasing emotional response to aversive images, focusing on the inferior frontal gyrus of the ventrolateral prefrontal cortex. The ventrolateral prefrontal cortex has been proposed as a key interface through which the neocortex exerts top-down control (ie, emotional regulation) over subcortical regions, such as the amygdala and nucleus accumbens, during the processing of emotionally salient experiences.^45,53^ To evaluate intervention effects on emotional regulation–related neural activity, we explored pairwise comparisons of each participant’s pretraining and posttraining scan activation patterns (Figure 3C). Positive *z* scores signify areas of increased emotional regulation–related activity after training in the intervention group.

## Results

### Participants

We randomized 21 PGY-1 surgery residents (8 [38%] women) using Wesleyan University’s Research Randomizer^54^ to either the modMBSR arm (n = 11; 4 [36%] women) or control arm (n = 10; 4 [40%] women), blocking for sex and surgical subspecialty designation. A participant assigned to the control group mistakenly attended the first modMBSR session, resulting in final participation and analysis of modMBSR (n = 12; 5 [42%] women) and control (n = 9; 3 [33%] women) (Table 1 and Figure 1). All participants were right-handed except for an ambidextrous participant in the modMBSR arm and a left-handed participant in the control arm.

**Table 1.** Demographic Characteristics of Study Sample

Characteristic	No. (%)	
	modMBSR Cohort (n = 12)^a^	Control Cohort (n = 9)^a^
Age, mean (SD), y	29.0 (2.4)	27.4 (2.1)
Sex		
Men	7 (58)	6 (67)
Women	5 (42)	3 (33)
Race^b^		
White	7 (58)	4 (44)
Black	0	1 (11)
Asian American	5 (42)	4 (44)
Subspecialty		
General surgery		
Categorical	4 (33)	1 (11)
Preliminary	1 (8)	1 (11)
Urology	1 (8)	1 (11)
Otolaryngology	1 (8)	1 (11)
Neurosurgery	1 (8)	0
OMFS	1 (8)	2 (22)
Plastics	1 (8)	1 (11)
Ophthalmology	1 (8)	1 (11)
Orthopedics	1 (8)	1 (11)

Abbreviations: modMBSR, modified mindfulness-based stress reduction; OMFS, oromaxillofacial surgery.

^a^ Groups as randomized were modMBSR (n = 11) and control (n = 10). One participant (a female, white, categorical general surgery resident) inadvertently attended the wrong first class and was therefore transferred to the modMBSR group.

^b^ No participants of Hispanic or other race/ethnicity were enrolled.

### Psychological Well-being and Distress

As shown in Table 2, mean group scores for perceived stress increased twice as much for the control arm as for the modMBSR arm at T2 (mean [SD] difference: modMBSR, 1.42 [5.74]; control, 3.44 [6.71]; η^2^ = 0.07) and decreased for both groups at T3 (mean [SD] difference: modMBSR, 1.00 [4.18]; control, 1.33 [4.69]; η^2^ = 0.09). Mean group scores for mindfulness increased twice as much in modMBSR participants as in control participants at T2 (mean [SD] difference: modMBSR, 3.08 [3.63]; control, 1.56 [4.28]; η^2^ = 0.13) and remained essentially stable, whereas control scores decreased slightly at T3 (mean [SD] difference: modMBSR, 2.17 [3.66]; control, −0.11 [6.19]; η^2^ = 0.15). Differences in mean group scores for trait resilience and grit were small and reflect small increases for the modMBSR arm and small decreases for the control arm at T2. At T3, we observed small increases for the modMBSR arm and stable scores for the control arm. Mean group scores for burnout increased in both groups at T2 and T3 (mean [SD] difference at T2: modMBSR, 4.50 [9.08]; control, 3.44 [6.71]; η^2^ = 0.01; mean [SD] difference at T3: modMBSR, 5.50 [9.96]; control, 5.56 [9.69]; η^2^ = 0.01) and varied little between groups. For symptoms of depression, mean group score for control participants increased more than twice as much as for modMBSR participants at T2, but at T3, scores decreased for both groups while remaining elevated above baseline.

**Table 2.** Multivariate Analysis of Treatment Effects on Well-being^a^ and Performance Outcomes^b^

Outcome; Instrument	Mean (SD)			ANCOVA^c^	Mean (SD)		ANCOVA^c^
	T1	T2	T2 − T1		T3	T3 − T1	
Perceived stress; PSS-10							
modMBSR	10.17 (4.41)	11.58 (5.44)	1.42 (5.74)	12.36	11.17 (3.69)	1.00 (4.18)	11.70
Control	13.22 (5.56)	16.67 (7.78)	3.44 (6.71)	15.63	14.56 (4.13)	1.33 (4.69)	13.84
* P* value	.18	.09	.47	.25	.06	.87	.19
Partial η^2^^d^	NA	NA	NA	0.07	NA	NA	0.09
Mindfulness; CAMS-R							
modMBSR	28.00 (4.09)	31.08 (3.61)	3.08 (3.63)	30.63	30.17 (4.59)	2.17 (3.66)	29.80
Control	25.89 (4.40)	27.44 (4.33)	1.56 (4.28)	28.05	25.78 (4.49)	−0.11 (6.19)	26.27
* P* value	.27	.05	.39	.11	.04	.30	.09
Partial η^2^^d^	NA	NA	NA	0.13	NA	NA	0.15
Resilience; ER89-10							
modMBSR	31.33 (5.11)	32.58 (5.44)	1.25 (3.02)	32.62	32.75 (5.59)	1.42 (2.50)	32.79
Control	31.44 (3.40)	30.89 (2.89)	−0.56 (2.83)	30.84	31.33 (3.12)	−0.11 (2.98)	31.28
* P* value	.96	.41	.18	.18	.50	.22	.22
Partial η^2^^d^	NA	NA	NA	0.10	NA	NA	0.08
Grit; Grit-S							
modMBSR	3.61 (0.72)	3.83 (0.67)	0.23 (0.52)	3.82	3.71 (0.62)	0.10 (0.51)	3.70
Control	3.57 (0.50)	3.57 (0.59)	0.00 (0.50)	3.58	3.53 (0.58)	−0.04 (0.51)	3.54
* P* value	.90	.36	.33	.27	.51	.53	.45
Partial η^2^^d^	NA	NA	NA	0.06	NA	NA	0.03
Burnout; aMBI							
modMBSR	23.92 (6.83)	28.42 (7.65)	4.50 (9.08)	27.71	29.42 (8.48)	5.50 (9.96)	28.69
Control	25.33 (7.62)	29.67 (5.90)	4.33 (7.78)	28.30	30.89 (8.57)	5.56 (9.69)	29.50
* P* value	.66	.69	.97	.82	.70	.99	.82
Partial η^2^^d^	NA	NA	NA	0.01	NA	NA	0.01
Depression; PHQ-9							
modMBSR	1.67 (1.56)	2.58 (2.61)	0.92 (3.03)	2.62	2.25 (2.34)	0.58 (3.15)	2.30
Control	0.89 (0.93)	3.33 (3.61)	2.44 (3.84)	3.29	2.56 (2.13)	1.67 (2.00)	2.48
* P* value	.20	.59	.32	.65	.76	.38	.87
Partial η^2^^d^	NA	NA	NA	0.01	NA	NA	<0.01
Working memory; NIH-EXAMINER							
modMBSR	1.04 (0.56)	1.39 (0.55)	0.35 (0.60)	1.39	1.73 (0.54)	0.68 (0.69)	1.76
Control	1.03 (0.52)	1.24 (0.49)	0.21 (0.74)	1.24	1.29 (0.50)	0.26 (0.58)	1.37
* P* value	.95	.51	.64	.52	.08	.16	.08
Partial η^2^^d^	NA	NA	NA	0.02	NA	NA	0.20
Executive composite; NIH-EXAMINER							
modMBSR	1.74 (0.42)	1.86 (0.43)	0.12 (0.41)	1.79	2.04 (0.34)	0.30 (0.51)	2.00
Control	1.46 (0.63)	1.61 (0.51)	0.15 (0.39)	1.71	1.64 (0.48)	0.07 (0.52)	1.69
* P* value	.25	.25	.86	.63	.04	.59	.09
Partial η^2^^d^	NA	NA	NA	0.01	NA	NA	0.15
Cognitive control; NIH-EXAMINER							
modMBSR	1.73 (0.45)	1.88 (0.38)	0.15 (0.40)	1.88	1.80 (0.40)	0.07 (0.59)	1.80
Control	1.71 (0.73)	1.64 (0.60)	−0.07 (0.32)	1.65	1.45 (0.52)	−0.26 (0.53)	1.45
* P* value	.94	.28	.19	.12	.09	.19	.08
Partial η^2^^d^	NA	NA	NA	0.13	NA	NA	0.16
Fluency; NIH-EXAMINER							
modMBSR	1.52 (0.67)	1.27 (0.52)	−0.25 (0.79)	1.17	1.59 (0.53)	0.07 (0.70)	1.47
Control	0.97 (0.80)	1.02 (0.71)	0.05 (0.46)	1.16	1.29 (0.78)	0.32 (0.52)	1.46
* P* value	.10	.36	.32	.94	.30	.38	.95
Partial η^2^^d^	NA	NA	NA	<0.01	NA	NA	<0.01
Peg transfer; FLS							
modMBSR	103.83 (34.57)	96.92 (20.46)	−6.92 (32.42)	100.82	102.42 (16.64)	−1.42 (29.79)	107.57
Control	129.00 (33.72)	122.44 (30.93)	−6.56 (29.25)	117.24	127.11 (41.11)	−1.89 (31.26)	120.25
* P* value	.11	.03	.98	.14	.07	.97	.30
Partial η^2^^d^	NA	NA	NA	0.11	NA	NA	0.06
Circle cutting; FLS							
modMBSR	176.58 (69.51)	152.50 (49.77)	−24.08 (63.00)	156.55	171.75 (71.96)	−4.83 (77.94)	173.40
Control	226.11 (102.16)	221.89 (65.19)	−4.22 (112.94)	216.49	237.78 (88.80)	11.67 (145.17)	235.58
* P* value	.20	.01	.61	.03	.08	.74	.11
Partial η^2^^d^	NA	NA	NA	0.23	NA	NA	0.13

Abbreviations: aMBI, abbreviated Maslach Burnout Inventory; ANCOVA, analysis of covariance; CAMS-R, Cognitive Affective Mindfulness Scale–Revised; ER89-10, Ego Resilience Scale, 10-item; FLS, Fundamentals of Laparoscopic Surgery; Grit-S, Short Grit Scale; modMBSR, modified mindfulness-based stress reduction; NA, not applicable; NIH-EXAMINER, National Institutes of Health Executive Abilities: Measures and Instruments for Neurobehavioral Evaluation and Research; PHQ-9, 9-item Patient Health Questionnaire; PSS-10, 10-item Perceived Stress Scale; T1, baseline (before start of residency); T2, postintervention (3.5 months after baseline); T3, end of year (12 months after baseline).

^a^ On the PSS-10, a higher score indicates more stress. On the CAMS-R, a higher score indicates greater mindfulness. On the ER89-10, a higher score indicates greater resilience. On the Grit-S, a higher score indicates more grit. On the aMBI, a higher score indicates more burnout. On the PHQ-9, a higher score indicates more or greater frequency of depressive symptoms.

^b^ For working memory, executive composite, cognitive control, and fluency, higher scores indicate greater executive function capacity. For peg transfer and circle cutting, lower scores indicate faster and more accurate motor skills.

^c^ Analysis of covariance shows variance between mean group score changes from T1 to T2 and from baseline T1 to T3. This method of analysis accounts for the effects of differences in treatment (ie, modMBSR vs control) as well as differences in baseline scores.

^d^ Partial η^2^ is the index of effect size for ANCOVA analyses, with 3 suggested cutoff points: small, less than 0.06; medium, 0.06 to 0.14; and large, greater than 0.14. For reference, partial η^2^ = 0.17 is approximately equivalent to Cohen *d* = 0.91, which is considered a large effect size.

### Changes in Executive Function

As shown in Table 2, mean group scores for executive composite and working memory increased more for participants in the modMBSR arm (executive composite: mean [SD] difference, 1.74 [0.42]; working memory: mean [SD] difference, 0.35 [0.60]) than control participants at T2 (executive composite: mean [SD] difference, 1.46 [0.63]; η^2^ = 0.01; working memory: mean [SD] difference, 0.21 [0.74]; η^2^ = 0.02). Mean group scores for cognitive control increased slightly in the modMBSR arm (mean [SD] difference, 0.15 [0.40]) but decreased slightly in the control arm (mean [SD] difference, −0.07 [0.32]; η^2^ = 0.13) at T2. At T3, mean group scores for working memory increased more than twice as much in the modMBSR arm (mean [SD] difference, 0.68 [0.69]) as in the control arm (mean [SD] difference, 0.26 [0.58]; η^2^ = 0.20), mean group scores for executive composite increased in the modMBSR arm (mean [SD] difference, 2.04 [0.34]) but decreased in the control arm (mean [SD] difference, 1.64 [0.48]; η^2^ = 0.20), and mean group scores for cognitive control decreased nearly 3-fold as much in the control arm (mean [SD] difference, −0.26 [0.53]) as in the modMBSR arm (mean [SD] difference, 0.07 [0.59]; η^2^ = 0.16). Fluency scores at T2 and T3 differed little between groups.

### Motor Skills Performance

The modMBSR group was faster in both the peg-transfer and circle-cutting tasks, accounting for variance in baseline scores. These relative effects persisted throughout the year, although times for both groups slowed from T2 to T3 (Table 2). For example, mean (SD) circle-cutting time improved more among participants in the modMBSR arm than in the control arm at T2 (modMBSR: −24.08 [63.00] seconds; control: −4.22 [112.94] seconds; η^2^ = 0.23) and at T3 (modMBSR: −4.83 [77.94] seconds; control: 11.67 [145.17] seconds; η^2^ = 0.13).

### Activation Patterns on fMRI During Emotional Regulation Task

Group results from whole-brain analysis are presented as raw *z* statistical maps to show that the patterns of task activation were robust and regionally specific (Figure 3A-C). Task validity was seen with ROI analysis based on an inferior occipital gyrus mask (Figure 3A, right panel), which showed greater activation of this visual processing region in response to negative images compared with neutral images without significant intergroup difference. Task fidelity was seen with ROI analysis based on an inferior frontal gyrus mask (Figure 3B, right panel), which showed statistically significant activation of this ventrolateral prefrontal cortex–associated region during the act of decreasing emotional response to negative images compared with simply viewing negative images, without significant intergroup difference.

Regarding intervention effects, the whole-brain analysis showed 2 primary areas of robust, regionally specific, and unique activation: the dorsal lateral prefrontal cortex (DL-PFC; centered on medial frontal gyrus and superior frontal gyrus) and the precuneus/posterior cingulate cortex. As expected, these did not reach voxelwise significance on whole-brain analysis in our small sample, but the more focused ROI analysis (Figure 3C, lower panel) confirmed statistically significant higher activation in all 3 areas (medial frontal gyrus, superior frontal gyrus, precuneus/posterior cingulate cortex), unique to the act of emotional regulation and only seen in the postintervention modMBSR group.

## Discussion

The clear need for enhanced stress resilience in surgery led us to conduct a pilot randomized clinical trial of modMBSR in PGY-1 surgery residents that was found to be feasible and acceptable.^34^ The preliminary evidence of efficacy regarding improved well-being and performance supports our outcome measures as sensitive and relevant to this population and suggests that our study methods can be successfully translated to an adequately powered future trial, as per the National Institutes of Health Stage Model for behavioral intervention development.^35^ We found increased mindfulness and less stress in participants in the modMBSR arm compared with those in the control arm, associated with medium to large effect sizes. We also found medium to large effect sizes associated with increased WMC among modMBSR participants and a marked decrement in cognitive control among control participants. Together with our finding of unique activation in brain regions associated with executive function and emotional regulation among modMBSR participants, our results suggest a sound working conceptual model and an intervention with potentially powerful effects.

Our finding that formal mindfulness training may improve aspects of well-being and distress is supported by our observation that mindfulness scores increased twice as much in the modMBSR arm as in the control arm, whereas stress and depressive symptoms scores increased twice as much in the control arm as in the modMBSR arm. These results were associated with medium to large effect sizes and echo statistically significant improvements in mood, affect, professional satisfaction, perceived stress, and protection from depressive symptoms seen with the use of MBIs in other groups.^55,56^ Relatedly, Sen et al^57^ showed a greater than 6-fold increase in depression during the intern year, with subsequent work demonstrating that higher perceived stress^58^ and lower perceived well-being^59^ predicted this evolution. Our results support the possibility that MBIs mitigate this risk through lowering perceived stress and increasing perceived well-being.

In contrast, our finding of increased burnout scores in both modMBSR and control participants, with little difference between the 2 groups and a small effect size, suggests the intervention does not influence burnout as measured and manifested in this population or simply reflects the underpowered nature of this study. While reports of the effect of MBIs on burnout are mixed,^7,8,16^ subdomain analysis of burnout (ie, emotional exhaustion vs depersonalization) in a larger sample may reveal effects that our small sample size precludes.

Our finding that modMBSR may improve or protect aspects of executive function is supported by increases in the executive composite and WMC scores in the modMBSR arm and marked decrement in the cognitive control factor score among control participants. These findings were associated with large effect sizes over time and merit further study. Executive function subserves decision making, problem solving, and the execution of complex procedures—skills clearly involved in surgical judgment and obviously valuable for physicians. The striking decrement in cognitive control scores within the control group raises concern that the rigors of residency training may cause relative cognitive impairment over time, while the lack of such a decrement in the modMBSR group suggests that formal mindfulness training may be protective. Prolonged stress in medical students has been associated with diminished performance on tests of executive function,^60^ whereas predeployment Marines have shown decrements in WMC that MBI training protected against.^17^

The faster peg-transfer and circle-cutting times for modMBSR participants vs control participants may be explained by the purported influence of MBI on attention and emotional regulation, cognitive skills increasingly recognized as critical for motor-skill performance.^61,62,63,64,65,66^ These results were associated with medium to large effect sizes and are supported by findings of a 2017 randomized clinical trial in general surgery residents,^67^ where performance training that included an emotional regulation component (eg, modulating self-criticism and positive self-talk) resulted in significantly higher scores on Fundamentals of Laparoscopic Surgery modules and higher-rated surgical skills.

Finally, our finding of greater activation in brain regions associated with executive function and self-awareness in modMBSR participants during an emotional regulation task is supported by the enhanced activity in the DL-PFC and precuneus/posterior cingulate cortex.^68,69,70^ These findings suggest that modMBSR affects changes at the level of neural substrates, which manifest in the setting of emotional stress. If increased activity correlates with enhanced capability in these parts of the brain, it could have broad implications for the benefit of MBIs. Clinical reasoning has been shown to decrease in internists with high burnout and concomitant deactivation of the DL-PFC,^71^ raising the possibility that increased activation in the DL-PFC following modMBSR may translate to improved clinical performance. Similarly, spatially complex bimanual coordination has been shown to be associated with significant activation of the precuneus, which is thought to mediate the interface between cognition and action in complex motor function.^72^

### Limitations

Study limitations include the small sample size, use of volunteers, and execution at a single institution with its own unique issues and resources. Our findings, while theoretically exciting, should be interpreted cautiously. Likewise, the ability to translate our intervention, study methods, and related findings to larger groups or other centers is promising but remains to be seen. Nevertheless, our findings support the value of further work to fully understand and more clearly demonstrate the association of MBIs with well-being and performance suggested here.

## Conclusions

The preliminary evidence presented here, supporting the potential benefits of MBIs for surgeons in the critical areas of well-being and executive function, justifies further exploration in a larger randomized clinical trial. Addressing perceived stress within surgical trainees may yield myriad downstream benefits, as seen in other populations. This characteristic of MBIs may provide a versatility otherwise lacking in well-being interventions and may affect key issues (eg, impaired mental health, cognition, and performance) that have otherwise been difficult to address. Moreover, as an internalized skill focused on perception and not just circumstance, mindfulness-based cognitive training has the potential to evolve and remain relevant across a surgeon’s career trajectory and life, making MBIs a promising means to address Accreditation Council for Graduate Medical Education programming mandates for physician trainee well-being.

## Notes

Supplement 1. **Trial Protocol**

Supplement 2. **eAppendix 1.** Emotional Regulation Task Data Acquisition **eAppendix 2.** fMRI Data Preprocessing **eReferences**

Supplement 3. **Data Sharing Statement**
