# Supplementary material for: Efficacy of Mindfulness-Based Cognitive Training in Surgery: Additional Analysis of the Mindful Surgeon Pilot Randomized Clinical Trial
Source: JAMA Netw Open. 2019 May 24;2(5):e194108. doi: 10.1001/jamanetworkopen.2019.4108 (PMC6632137; doi:10.1001/jamanetworkopen.2019.4108)
Supplement: Supplement 1. — Trial Protocol [file jamanetwopen-2-e194108-s001.pdf]

Study Protocol and Statistical Analysis Plan

Mindful Mental Training for Surgeons to Enhance Resilience and Performance under Stress ("Mindful Surgeon")

ClinicalTrials.gov Identifier: NCT03141190

Recruitment Status: Completed

Sponsor: University of California, San Francisco

Information provided by (Responsible Party):

Carter Lebares, University of California, San Francisco

Study Description

Brief Summary:

Background:

Burnout and overwhelming stress are growing issues among surgeons and are associated with mental illness, attrition and diminished patient care. Among surgical trainees, burnout and distress are alarmingly prevalent but high inherent mindfulness has been shown to decrease the risk of depression, suicidal ideation, burnout and overwhelming stress by more than 75%. In other high-stress populations formal mindfulness training has been shown to improve mental health and buffer overwhelming stress and yet this approach has not been tried in surgery.

The aim of this study is to evaluate feasibility and acceptability of modified mindfulness-based stress reduction (MBSR) training among PGY-1 surgery residents and to obtain initial evidence of efficacy in regard to well-being and performance.

Design: A pilot randomized clinical trial of modified MBSR versus an active control.

Setting: Residency training program, tertiary academic medical center.

Participants: PGY-1 surgery residents.

Intervention: Weekly two-hour modified MBSR classes (compared to an active control) and 20 minutes of suggested daily home practice over an eight-week period.

Main Outcomes and Measures:

Primary outcome is feasibility, assessed along six domains (demand, implementation, practicality, acceptability, adaptation and integration), using focus groups, interviews, surveys, attendance, daily practice time and subjective self-report of experience.

Secondary outcomes include perceived stress, mindfulness and executive function (specifically working memory capacity), followed by psychosocial well-being (burnout, depression, resilience), performance (motor skills testing) and functional brain scans focused on areas associated with reappraisal as a surrogate for emotional control.

This study seeks to demonstrate the feasibility of mindfulness training in surgery PGY-1s while simultaneously providing preliminary quantitative data on the effects of mindfulness training in a randomized, controlled setting. Data will inform modifications to the MBSR curriculum that enhance feasibility and inform sample size calculations for subsequent, adequately-powered RCTs which will likely need to be multi-center trials.

Results could potentially impact formal medical training, the mental health of providers at every level, and the overall quality of patient care.

Condition or disease	Intervention/treatment
Burnout Syndrome<br>Surgery<br>Stress	Behavioral: Mindfulness Based Stress Reduction - modified<br>Behavioral: Active listening and reading

Detailed Description:

Mounting evidence shows that burnout, a critical metric for dissatisfaction and distress, is a growing problem within medicine. Burnout is a syndrome associated with worse physician performance, patient outcomes, and hospital economics. The quadruple aim of healthcare underscores that physician fulfillment is a critical part of any sustainable reform and appropriately frames physician burnout and fulfillment as issues that impact everyone, not just individual providers.

Burnout is believed to arise from a mismatch between expectations and reality, with more than half of practicing physicians and trainees reported to suffer from this problem. Among general surgery residents, the prevalence of burnout is estimated at 69% and dramatically increases the odds of both overwhelming stress and distress symptoms. The relationship between overwhelming stress and burnout is particularly concerning because extensive evidence links overwhelming stress to detrimental effects on learning, memory, decision-making, and performance.

A recent meta-analysis suggests that stress management/mindfulness interventions are particularly effective at addressing burnout on the individual level. Small cohort studies and controlled trials have shown mindfulness-based interventions to be effective at reducing stress and burnout in medical students, primary care physicians, internists, and other healthcare providers.

In general surgery trainees, inherent mindfulness tendencies (shown to increase following mindfulness training), decrease the risk of burnout, overwhelming stress, and distress symptoms by 75% or more. This suggests that mindfulness tendencies may already be used, albeit unconsciously, to cope within the high-stress culture of surgery. Indeed, isolated studies of performance strategies that involve emotional regulation, and focused attention (qualities shared with mindfulness training) have also demonstrated improvements in surgeons' technical performance and perceived stress.

Mindfulness meditation training involves the cultivation of moment-to-moment awareness of thoughts, emotions and sensations (also known as interoception), the development of non-reactivity in response to stimuli (also known as emotional regulation), and the enhancement of perspective-taking regarding oneself and others. The most scientifically studied form of mindfulness training is the secular Mindfulness-Based Stress Reduction (MBSR) developed by Jon Kabat-Zinn in the 1970s. MBSR is formally trained through an eight-week codified curriculum and has been shown to decrease stress and burnout, protect executive function, and enhance performance in multiple high-stress populations.

In spite of such evidence, mindfulness training among surgeons has only occasionally been suggested or informally pursued, partly due to a disconnect between the indefatigable stoicism of surgery and mindfulness, which is often perceived as relaxation rather than a skill to enhance resilience. Moreover, the time pressures of surgical training make additional responsibilities and new curricula seem impossible.

In fact, the global effects of mindfulness training, as opposed to other interventions that target a single outcome, may prove to be its biggest asset. Individuals don't manifest the effects of overwhelming stress and burnout in identical ways, making an up-stream intervention with myriad downstream effects the most efficient method for intervening on large, diverse populations. Moreover, while other forms of skills training or mental health interventions require recurrent time away from work, mindfulness training involves an initial investment of time but then can be strengthened through practice in everyday settings - within the daily life, not separate from it.

To systematically examine the feasibility of formal mindfulness training during surgery internship at a tertiary academic center, we undertook the "Mindful Surgeon" pilot study. Our secondary goal is to gather preliminary evidence of efficacy, to guide future design of a scalable, adequately powered trial.

Study Design

Study Type:	Interventional
Actual Enrollment:	21 participants
Allocation:	Randomized
Intervention Model:	Parallel Assignment<br><br>Randomized, partially-blinded
Masking:	Double (Participant, Outcomes Assessor)<br><br>Participants do not know we are testing mindfulness only that they will be learning stress-reduction skills for surgeons
Primary Purpose:	Prevention
Official Title:	Mindfulness Training to Improve Mental Health, Stress and Performance In Physicians
Actual Study Start Date:	June 2016
Actual Primary Completion Date:	December 31, 2017
Actual Study Completion Date:	December 31, 2017

Arms and Interventions

Arm	Intervention/treatment
Experimental: Mental Training for Surgeons<br><br>Mindfulness-Based Stress Reduction (MBSR, as published elsewhere extensively) slightly modified by shortening the eight weekly classes to 2 hours each and the home practice requirement to 20 minutes. Taught by a veteran MBSR teacher with greater than 10,000 hours of personal practice and nearly 10 years of formal MBSR teaching experience.	Behavioral: Mindfulness Based Stress Reduction - modified<br><br>already described<br><br>Other Names:<br><br>• MBSR
Active Comparator: The Mind of a Surgeon<br><br>8 weekly classes of 2 hours each with group reading and discussion of selected articles and	Behavioral: Active listening and reading<br><br>group reading, listening and

stories about the ethos and experience of becoming a surgeon. Designed and administered by a surgical faculty member with extensive experience in surgical education and scholarly work in the area of the 'surgical personality'.	discussion of articles pertaining to the development and experience of the surgical personality

Outcome Measures

Primary Outcome Measure:

1. Change in Stress [Time Frame: baseline, 8wks (post-intervention),12-month follow-up]

    Cohen's Perceived Stress Scale (PSS)

Secondary Outcome Measures:

1. Change in Executive Function [Time Frame: baseline, 8wks (post-intervention),12-month follow-up]

    Executive function as assessed via working memory capacity, cognitive control and executive composite components of the NIH EXAMINER battery.

Other Pre-specified Outcome Measures:

1. Change in Motor skills [Time Frame: baseline, 8wks (post-intervention),12-month follow-up]

    Performance as assessed by the Fundamentals of Laparoscopic Surgery (FLS) modules

2. Change in Functional neuroanatomic changes [Time Frame: baseline, 8wks (post-intervention),12-month follow-up]

    Functional changes in areas associated with reappraisal/emotional regulation (amygdala, hippocampus, reward circuitry, appraisal

pathway) as evidenced by fMRI BOLD and DTI brain scans analyzed by whole brain and a prior region of interest approaches.

3. Change in Psychological well-being [Time Frame: baseline, 8wks (post-intervention),12-month follow-up]

   Burnout (Maslach burnout inventory), depression (PHQ-9), resilience (ER89), Grit (GRIT-S), mindfulness (CAMS-R)

Eligibility Criteria

Ages Eligible for Study:	18 Years and older
Sexes Eligible for Study:	All
Gender Based:	No
Accepts Healthy Volunteers:	Yes

Criteria

Inclusion Criteria:

- UCSF surgical interns entering training. Do not meet exclusion criteria.

Exclusion Criteria:

- Current personal mindfulness practice, pregnancy, breast-feeding or implanted MRI-incompatible metal.

Contacts and Locations

Locations

United States, California

University of California San Francisco

San Francisco, California, United States, 94143

Investigators

Principal Investigator:	Carter Lebares, MD	University of California, San Francisco

More Information

Responsible Party:	Carter Lebares, Assistant Professor of Surgery In Residence, University of California, San Francisco
ClinicalTrials.gov Identifier:	NCT03141190
Other Study ID Numbers:	16-19688
Last Verified:	September 2018

Individual Participant Data (IPD) Sharing Statement:

Plan to Share IPD: No

Human Subjects Protection Review Board Status: Approved

Studies a U.S. FDA-regulated Drug Product: No

Studies a U.S. FDA-regulated Device Product: No

Statistical Analysis Plan

All analyses will be conducted on the intent-to-treat sample. Outcomes (from questionnaires, tests and assays) will be summarized for each group at 3 time points. I will test for inter-group differences (using independent sample t-tests and Pearson's chi- square tests). I will conduct analyses to serve as preliminary data for the design of afuture RCT of efficacy. Linear mixed-effects modeling (ANCOVA) will be used for multivariate analysis, adjusting for baseline variation and calculating effect sizes.
