# Supplementary material for: Efficacy of Mindfulness-Based Cognitive Training in Surgery: Additional Analysis of the Mindful Surgeon Pilot Randomized Clinical Trial
Source: JAMA Netw Open. 2019 May 24;2(5):e194108. doi: 10.1001/jamanetworkopen.2019.4108 (PMC6632137; doi:10.1001/jamanetworkopen.2019.4108)
Supplement: Supplement 2. — eAppendix 1. Emotional Regulation Task Data Acquisition eAppendix 2. fMRI Data Preprocessing eReferences [file jamanetwopen-2-e194108-s002.pdf]

Supplementary Online Content

Lebares CC, Guvva EV, Olaru M, et al. Efficacy of mindfulness-based cognitive training in surgery: additional analysis of the Mindful Surgeon pilot randomized clinical trial. *JAMA Netw Open*. 2019;2(5):e194108. doi:10.1001/jamanetworkopen.2019.4108

eAppendix 1. Emotional Regulation Task Data Acquisition
eAppendix 2. fMRI Data Preprocessing
eReferences

This supplementary material has been provided by the authors to give readers additional information about their work.

© 2019 Lebares CC et al. *JAMA Network Open.*

eAppendix 1.

Emotional Regulation Task Data Acquisition. Whole-brain fMRI data were acquired on a Siemens 3T Prisma fit scanner system with a 64-channel head coil (Siemens Healthcare). Anatomic images were acquired with a T1 mprage sequence with a TR of 2300ms, TE of 2.9ms, flip angle of 9, FOV of 256mm, 160 slices and 1.0x1.0x1.0mm voxel size. Images for both resting state (8:05min) and the emotion-regulation/reappraisal task (5:14min for each of three runs), were acquired with an EPI sequence with a TR of 850ms, TE of 32.8ms, flip angle of 45, FOV of 211mm, 66 slices and 2.2x2.2x2.2mm voxel size. The task and associated stimulus images were presented using E-Prime software (PST Inc.). Images were displayed on a monitor in the scanner suite and were viewable through a system of back-projecting mirrors mounted to the head coil unit. For each image trial, participants viewed a fixation cross (jittered 3-5 seconds), the instruction ‘LOOK’ or ‘DECREASE’ (1.5 seconds), the negative or neutral image (8 seconds), and instructions to ‘now rate the intensity of your emotional experience’ in regard to the image (1.5 seconds), a button box was used to respond.

© 2019 Lebares CC et al. *JAMA Network Open.*

eAppendix 2

fMRI Data pre-processing. Analysis methods were performed using FSL 5.0 (FMRIB Software Library, Oxford University)[1] and Freesurfer 6.0 (Athinoula A. Martinos Center for Biomedical Imaging, Harvard-MIT, Boston).[2] First, the data was skull-stripped by removing signal from non-brain tissues using mri_convert utility for the 3D T1-weighted images, and BET (Brain Extraction Tool)[3] for the T2* sensitive images. Each individual echo planar imaging (EPI) time-series was motion corrected to the middle time point using a 6 parameter, rigid-body method (implemented in MCFLIRT).[4] The data then underwent highpass temporal filtering (100s cutoff) and were smoothed using a Gaussian kernel of 5mm FWHM (full width half maximum). Autocorrelation was corrected with a pre-whitening technique, and standard motion parameters were included as nuisance regressors (within Feat; fMRI Expert Analysis Tool).[5] Each trial type was then modeled as a separate condition using a double-gamma hemodynamic response convolution function with a temporal derivative.

© 2019 Lebares CC et al. *JAMA Network Open.*

eReferences.

1. Smith SM, Jenkinson M, Woolrich MW, et al. Advanced in functional and structural MR image analysis and implementation as FSL. *Neuroimage*. 2004;23: Suppl 1:S208-S219.
2. Flandin G, Friston KJ. Statistical Parametric Mapping. *Scholarpedia*. 2008; 3(4):6232.
3. Fischl B, Salat DH, Busa E, et al. Whole brain segmentation: Automated labeling of neuroanatomical structures in the human brain. *Neuron*. 2002;33(3):341–355.
4. Jenkinson M, Bannister PR, Brady JM, Smith SM. Improved optimization for the robust and accurate linear registration and motion correction of brain images. *Neuroimage.* 2002;17:825-841.
5. Woolrich MW, Ripley BD, Brady JM, Smith SM. Temporal autocorrelation in univariate linear modelling of fMRI data. *Neuroimage*. 2001;14:1370-1386.

© 2019 Lebares CC et al. *JAMA Network Open.*
