# Supplementary material for: Efficacy of Mindfulness-Based Cognitive Training in Surgery: Additional Analysis of the Mindful Surgeon Pilot Randomized Clinical Trial
Source: JAMA Netw Open. 2019 May 24;2(5):e194108. doi: 10.1001/jamanetworkopen.2019.4108 (PMC6632137; doi:10.1001/jamanetworkopen.2019.4108)
Supplement: Supplement 3. — Data Sharing Statement [file jamanetwopen-2-e194108-s003.pdf]

Data Sharing Statement

Lebares. Efficacy of Mindfulness-Based Cognitive Training in Surgery. *JAMA Netw Open*. Published May 24, 2019.
10.1001/jamanetworkopen.2019.4108

Data
Data available: No

Additional Information
Explanation for why data not available: Due to the sensitive nature of the questions asked and evaluation performed in this study, participants were assured raw data would remain confidential and would not be shared.
